# Reproductive Toxicity of Nanomaterials Using Silver Nanoparticles and *Drosophila* as Models

**DOI:** 10.3390/molecules29235802

**Published:** 2024-12-09

**Authors:** Mohamed Alaraby, Doaa Abass, Javier Gutiérrez, Antonia Velázquez, Alba Hernández, Ricard Marcos

**Affiliations:** 1 Group of Mutagenesis, Department of Genetics and Microbiology, Faculty of Biosciences, Universitat Autònoma de Barcelona, Campus of Bellaterra, 08193 Cerdanyola del Vallès, Spain; doaa_abass69@yahoo.com (D.A.); javier.gutierrez.garcia@uab.cat (J.G.); antonia.velazquez@uab.cat (A.V.); 2 Zoology Department, Faculty of Science, Sohag University, Sohag 82524, Egypt

**Keywords:** AgNPs, ovary/testis internalization, gene expression changes, *Drosophila melanogaster*, reproductive toxicity

## Abstract

Reproductive toxicity is of special concern among the harmful effects induced by environmental pollutants; consequently, further studies on such a topic are required. To avoid the use of mammalians, lower eukaryotes like *Drosophila* are viable alternatives. This study addresses the gap in understanding the link between reproductive adverse outcomes and the presence of pollutants in reproductive organs by using *Drosophila.* Silver nanoparticles (AgNPs) were selected for their ease of internalization, detection, and widespread environmental presence. Both male and female flies were exposed to AgNPs (28 ± 4 nm, 100 and 400 µg/mL) for one week. Internalization and bioaccumulation of AgNPs in organs were assessed using transmission electron microscopy, confocal microscopy, and inductively coupled plasma mass spectrometry. Substantial accumulation of AgNPs in the gastrointestinal tract, Malpighian tubules, hemolymph, reproductive organs (ovaries and testes), and gametes were observed. The highest AgNP content was observed in testes. Exposure to AgNPs reduced ovary size and fecundity, though fertility and gender ratios of the offspring were unaffected. Significant deregulation of reproductive-related genes was observed, particularly in males. These findings underscore the utility of *Drosophila* as a model for evaluating reproductive hazards posed by AgNP exposure. The ease of AgNP internalization in *Drosophila* reproductive targets could be extrapolated to mammalians, raising concerns about the potential impacts of nanoparticle exposure on reproduction toxicity in humans.

## 1. Introduction

According to the European Chemical Agency (ECHA), reproductive toxicity refers to the effects on fertility caused by xenobiotics, including environmental pollutants of emergent health concern. Such effects can result from exposures of either parent (before conception) or of the developing organism [1]. As a complex trait, the processes underlying reproductive toxicity necessitate continuous crosstalk between researchers and methodologies from different disciplines [2]. Focusing on the experimental approaches used to determine reproductive toxicity, it is alarming to note the large number of laboratory mammals involved in such research studies. Despite the EU REACH regulation indicating that mammals should only be used as a last resort, by the end of 2022, about 2.9 million laboratory animals were used to meet REACH demands in reproductive toxicity [3]. This underscores the urgent need for alternative models, with *Drosophila* emerging as a promising candidate.

*Drosophila* is an experimental model widely used in many research fields due to its numerous advantages. The *Drosophila* genome shares a high percentage (75%) of gene homology with genes involved in human pathologies [4]. Consequently, *Drosophila* has been proposed as a suitable model to understand the etiology of many human diseases [5], and it is considered a good model for extrapolating findings to humans, including reproductive toxicity effects [6,7].

Over time, many agents have been tested in *Drosophila* to determine harmful effects on reproductive traits. Surprisingly, few studies have focused on nanoparticles. Exposure to silver nanoparticles (AgNPs) in males has been shown to significantly increase reactive oxygen species (ROS) levels in the testes, reduce the number of germinal stem cells, and promote premature differentiation [8]. Additionally, adult exposure to AgNPs affects egg laying and impairs ovary growth. When larvae were exposed, reduced survival, longevity, ovary size, and egg-laying capability were observed, along with trans-generational effects [9]. Prolonged exposure to titanium dioxide nanoparticles (TiO_2_NPs) significantly impaired spermatid elongation in *Drosophila* testes, with RNA-seq analysis indicating 251 differentially expressed genes affecting multiple metabolic pathways, including carbohydrate metabolism and cytochrome P450 [10]. The reproductive toxicity of polystyrene nanoplastics (PS-NPLs) has also been evaluated showing the presence of PS-NPLs in the crop, gut, and ovaries; decreased egg production and hatching rate; and delayed development [11]. Transcriptomic analysis in that study revealed the differential expression of reproductive-related genes.

Given the advantages of *Drosophila* as an in vivo model and the limited studies on the hazardous effects of AgNPs on its reproductive potential, this study aims to employ a wide array of methodological approaches to determine how AgNP exposure affects different reproductive traits. AgNPs are widely used in consumer products and biomedical devices, with potential reproductive toxicity reported in mammalian males [12]. The key aspects of this study are the (i) development of a simple and novel method for adult exposure; (ii) exposure of both males and females with all mating combinations tested; (iii) quantification of Ag levels in males, females, testes, ovaries, and eggs with ICP-MS; (iv) determination of internalization in different organs using transmission electronic microscopy and confocal microscopy; and (v) assessment of changes in the expression of genes involved in reproductive functions. Linking reproductive toxic effects with the internalization of AgNPs in reproductive organs is one of the main aspects of this study.

## 2. Results and Discussion

### 2.1. Feeding/Exposure

Despite numerous studies on the reproductive toxicity of AgNPs, significant knowledge gaps remain, particularly regarding bioaccumulation, tracking pathways, well-designed mechanistic approaches, new toxic endpoints, sex differences, and molecular responses [13]. This study aims to address these issues using *Drosophila melanogaster* as a suitable in vivo model.

Given the distinct differences between the developmental stages (larvae and adults) in holometabolic insects, the standard corn meal medium diet for *Drosophila melanogaster* larvae is not suitable for adults, which have sucking and sponging mouthparts. The adult diet typically consists of a sugar/yeast food mixture that solidifies when prepared with agar [14]. Adult fruit flies feed on the surface of this food by extending their proboscis to draw it into their mouth [15]. In contrast, larvae live within the diet medium and are in direct contact with the agents mixed into it. Exposing adults to an aqueous diet, however, poses a risk of drowning [16]. Therefore, developing an effective adult exposure method is crucial before commencing experiments [15].

**Figure 1:**
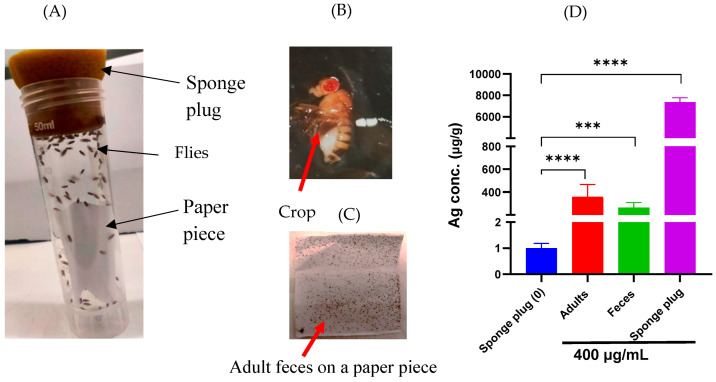
Experimental design and AgNP internalization quantification. (**A**) Flies were kept in 50 mL vials containing a small piece of paper and closed with a sponge plug. Flies were fed via a wetting sponge plug with 1 mL of peach–grape juice. Non-treated flies received only juice, while treated flies were exposed to AgNPs (100 and 400 µg/mL). (**B**) A photograph of a treated adult (as confirmed by the crop color). (**C**) Feces of the treated adults on the piece of paper. (**D**) ICP-MS analysis of silver in sponge plug wetted with juice (non-mixed or mixed with AgNPs), as well as in treated adult tissues and feces. Multiple pairwise comparisons (Turkey’s test) were performed using one-way ANOVA analysis. *** *p* < 0.001 and **** *p* < 0.0001.

To optimize dietary/exposure intake for adult *Drosophila*, we designed a systematic approach where flies were fed by wetting the sponge plugs of rearing vials with peach–grape juice. The validity of this feeding/exposure protocol was verified through ICP-MS analysis and morphological investigations. *Drosophila* adults were exposed to AgNPs mixed with peach–grape juice, a medium highly appealing to them (Figure 1A). Morphological investigation revealed AgNP–juice present in the crop area of the adult abdomen and in the feces, observed as black spots on the paper pieces (Figure 1B,C). ICP-MS analysis of the sponge plugs wetted with AgNP–juice mixture confirms high levels of Ag when compared to plugs wetted with juice alone (Figure 1D). These findings confirm the simplicity and effectiveness of our method for exposing *Drosophila* adults to AgNPs.

### 2.2. Internalization

The effects of nanoparticle (NP) exposure are contingent upon their ability to overcome biological barriers and distribute throughout the body, organs, tissues, and cells. NP internalization is complex, influenced by properties such as shape, size, surface chemistry, and stiffness [17]. The optimal NP size for rapid internalization is 27–30 nm, which aligns with the AgNPs (28 ± 4 nm) used in this study [17].

To investigate AgNP internalization and bioaccumulation, we employed various techniques, including ICP-MS, TEM, and confocal microscopy. Notably, most studies on *Drosophila* internalization have focused on larvae [18,19], with limited research on adults. This is due to the advantages of larvae like short life span, ease of feeding, voracity, and high activity. However, larvae are not suitable for studying reproduction and sex-related issues, which our adult feeding/exposure protocol addresses.

**Figure 2:**
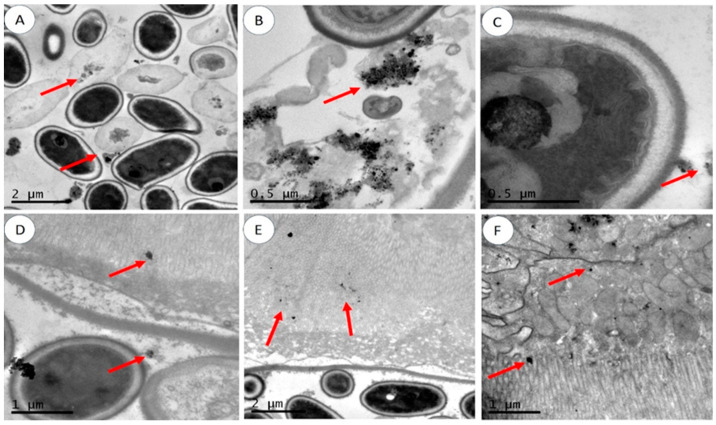
TEM images of the intestine of Drosophila. (**A**) AgNPs are distributed in the lumen, (**B**,**C**) attached to symbiotic bacteria, (**D**) closed to the peritrophic membrane, and (**E**,**F**) distributed inside enterocytes. Adults were treated with AgNPs, dissected, and investigated using TEM. Red arrows point out AgNPs.

**Figure 3:**
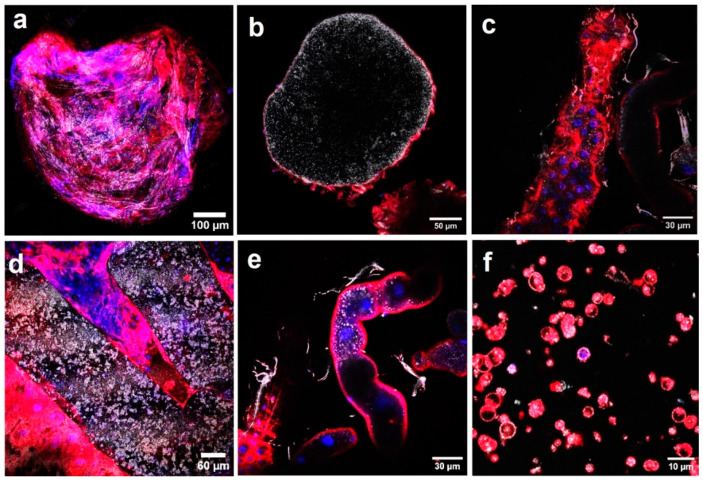
Confocal fluorescent images of different organs of *Drosophila* adults after AgNPs exposure. (**a**,**b**) Crop, (**c**,**d**) intestine, (**e**) Malpighian tubes, and (**f**) hemolymph with hemocytes. Adults were treated with AgNPs, dissected, and investigated with a confocal microscope.

TEM (Figure 2) and confocal microscopy (Figure 3) were used to examine AgNP presence in the intestinal tract. TEM revealed large patches of AgNPs in the intestines of flies exposed to 400 µg/mL AgNPs, distributed in the lumen (A), attached to symbiotic bacteria (B, C), near the peritrophic membrane (D), and within enterocytes (E, F). Confocal microscopy showed extensive AgNP distribution in the crop (a, b), intestines (c, d), Malpighian tubules (e), and hemolymph/hemocytes (f). Similar findings have been reported in other species, such as *Cyprinus carpio* and mice, indicating bioaccumulation and associated adverse effects [20,21].

**Figure 4:**
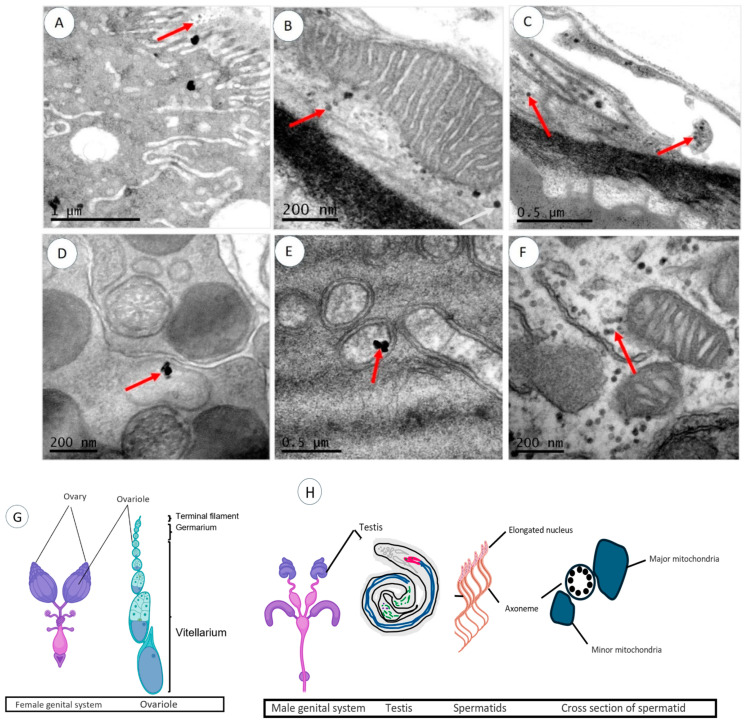
TEM images of the reproductive organs of Drosophila. (**A**–**C**) Ultrathin section of *Drosophila* ovaries showing the distribution of AgNPs inside germanium (**A**), close to mitochondria (**B**), and in outer membranes (**C**). (**D**–**F**) Ultrathin sections of *Drosophila* testis showing the distribution of AgNPs inside spermatid cyst (**D**,**E**) and surrounding mitochondria (**F**). (**G**,**H**) Schematic drawings of *Drosophila* female and male genital systems, respectively. Red arrows point out AgNPs.

**Figure 5:**
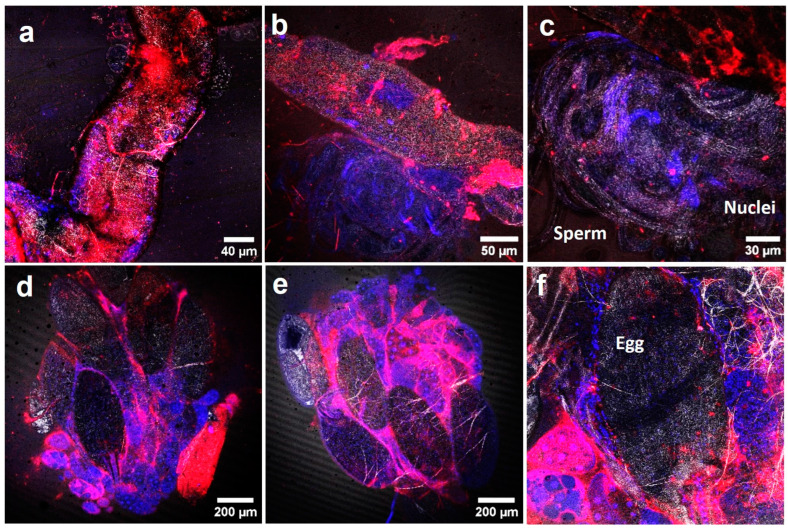
Confocal fluorescent images of AgNPs in different reproductive organs of *Drosophila* adults after one week of exposure. (**a**–**c**) Testis, (**d**–**f**) ovary. Adults were treated with AgNPs, dissected, and investigated by confocal microscopy.

TEM further confirmed AgNP presence in reproductive organs, including the ovaries and testes (Figure 4). AgNPs accumulated in the ovary’s germarium cells (A), attached to mitochondria (B), and in outer follicular epithelial cells (C). Confocal microscopy (Figure 5) also showed AgNP bioaccumulation in the testes (a, b), sperm (c), ovaries (d, e), and mature eggs (f). These findings suggest potential disturbances in spermatogenesis, oogenesis, and gene expression due to AgNP exposure [22].

Ultrastructural investigations have also demonstrated changes in other organisms exposed to AgNPs, such as beetles (*Blaps polychresta)* [23]. In rats, AgNP exposure reduced testicular weights, changed hormone levels, and interfered with sperm parameters [24]. In addition, AgNPs (10–72 nm) diffused into zebrafish embryos crossing the large pore size of the chorion [25], and embryos showed reduced yolk size and bend tails when exposed to high concentrations of AgNPs [26].

**Figure 6:**
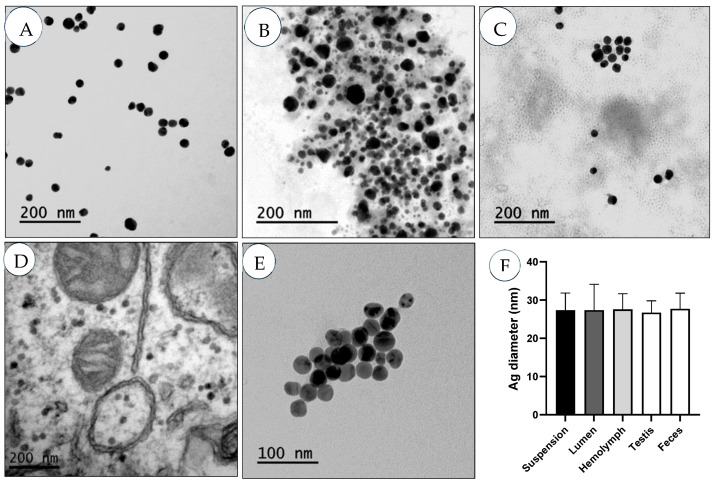
TEM representative images of AgNPs, whether in stock dispersion (**A**) or *Drosophila* adults’ tissues (**B**–**D**) after oral administration. AgNPs were determined in the intestine lumen (**B**), hemolymph (**C**), testes (**D**), or extracted from feces (**E**). AgNP diameters (**F**) were measured with the ImageJ program.

Once internalized, NP size can change due to factors such as the adsorption of proteins (protein corona) and the release of ions. The toxicity of AgNPs has been associated with the released ions, which compromise cell membrane integrity, causing cell necrosis and oxidative stress [27]. However, some studies postulated the resistance of nanoparticulated materials against dissolutions. In this context, no changes in AgNP diameter were observed after 7 months of aging [28], and it is important to note that protein-rich environments can lead to significant long-term nanoparticle stabilization [29]. To address this topic, AgNP diameters were measured in different scenarios (Figure 6), whether in juice “as primary size” (A) or inside the midgut lumen (B), hemolymph (C), testis (D), or feces (E). Remarkably, AgNP diameter remained unmodified in the evaluated scenarios, indicating AgNP stability. The role of the potential release of silver ions in the toxic effects of AgNPs was determined in a previous study, showing a low release ability (≈10%) without a relevant role of toxicity because similar effects were detected by comparing the effects of equal concentrations of AgNPs and AgNO_3_ in *Drosophila* [30].

**Figure 7:**
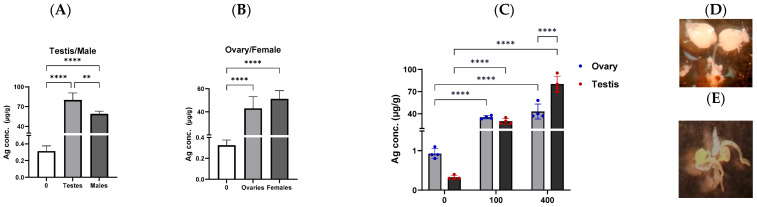
(**A**) Ag concentration in testes and (**B**) ovaries of adults exposed to 400 µg/mL AgNPs. (**C**) Ag concentration comparison between testes and ovaries of adults exposed to different Ag concentrations (**D**,**E**). Photographs of the ovary and testis, respectively. Multiple pairwise comparisons (Turkey’s test) were performed using one-way and two-way ANOVA, ** *p* < 0.01, and **** *p* < 0.0001.

**Figure 8:**
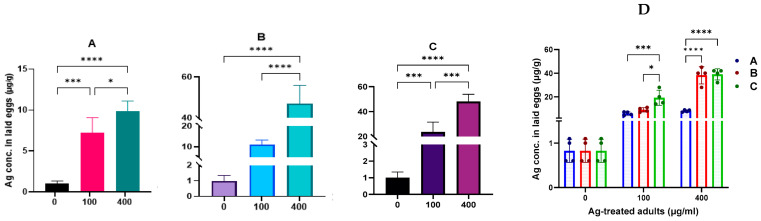
Ag concentration in eggs of *Drosophila* exposed to AgNPs compared to non-treated flies. Adults were exposed to AgNPs for one week and divided into three groups: (**A**) fed with non-treated juice one day before laying eggs, (**B**) fed with non-treated juice during laying eggs, and (**C**) not fed with juice during laying eggs. (**D**) Comparison between the three previous exposure conditions (A, B, C). * *p* < 0.05, *** *p* < 0.001, and **** *p* < 0.0001.

To quantify the Ag concentration in exposed flies, particularly in the reproductive organs and eggs, samples were analyzed using ICP-MS (Figure 7 and Figure 8). As expected, Ag concentrations significantly increased in males and their testes (59.0 ± 4.1 µg/g and 79.9 ± 10.7 µg/g, respectively) compared to unexposed adults (0.31 ± 0.06 µg/g) (Figure 7A). Notably, the Ag concentrations in testes were higher than in whole males, indicating specific bioaccumulation of AgNPs in the testes. Similar findings were observed in exposed females and their ovaries, with Ag contents significantly increasing to 51.3 ± 7.1 µg/g and 43.0 ± 10.1 µg/g, respectively, compared to unexposed adults (Figure 7B). However, no significant differences in bioaccumulated Ag were observed between females and their ovaries. When comparing Ag concentrations in testes and ovaries, the testes accumulated more Ag than the ovaries, proportional to AgNP exposure levels (100 or 400 µg/mL) (Figure 7C). There are limited data on Ag content in reproductive organs following AgNP exposure. However, studies have shown the presence of Ag in various body tissues (the lungs, liver, brain, and kidneys) of rat offspring from pregnant rats exposed to 250 mg/kg AgNPs (7.9 ± 0.95 nm), suggesting the possible transfer of Ag from pregnant dams to fetuses via the placenta [31].

The impact of AgNPs on embryos and offspring is not well studied. In mice, the effects of AgNPs on embryos were found to be size- and concentration-dependent, with particles smaller than 40 nm having a more significant impact on embryo development [32]. Similarly, exposure to 20–30 nm AgNPs at 200 mg/kg/day induced abnormalities in the morphology of rat sperm [33]. In our study, we aimed to determine whether bioaccumulated AgNPs in the reproductive organs could be transferred to the progeny. We measured the silver content in eggs after fly exposure to 100 and 400 µg/mL AgNPs (Figure 8). To assess the temporal change in Ag content in eggs post-exposure, adult flies were divided into three groups, as described in Section 3.5. The results showed that eggs retained significant levels of silver in a concentration-dependent manner. Interestingly, the amount of silver decreased over time after exposure ceased. This decrease was more pronounced in eggs laid by flies that were fed non-treated juice one day before laying eggs compared to the other two groups (D). Our findings provide a potential explanation for the transgenerational effects observed in *Drosophila* exposed to AgNPs, where growth and survival effects were evident in the F2 generation of exposed parents [9].

### 2.3. Ovary Size

**Figure 9:**
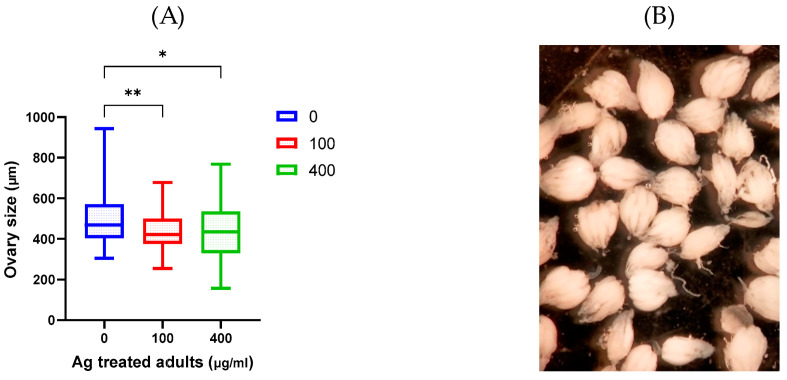
Ovaries size of *Drosophila melanogaster* exposed to AgNPs in comparison to non-treated flies (**A**). Photograph image showing dissected Drosophila ovaries (**B**). Multiple pairwise comparisons (Dunn’s test) were performed using Kruskal–Wallis. * *p* < 0.05 and ** *p* < 0.01.

The ovary is a vital organ for female reproduction. The main functions include sex steroid hormone synthesis, follicular development, and achieving oocyte meiotic and development competence for proper fertilization [34]. Because it was presumed that alterations in ovarian development/size might compromise fertility, this work aimed to evaluate if AgNP accumulation in ovaries compromises their size. This would result in adverse effects on female reproductive fitness. Samples of females whether unexposed or exposed to different AgNP concentrations were dissected and their ovaries were measured using the ImageJ program. The results demonstrated ovary atrophy, where their sizes were significantly decreased with exposure to AgNPs whether in low or high concentration (Figure 9). Our results agree with the size reduction reported in *Drosophila* ovaries exposed to AgNPs (250 mg/L) [9]. Since insulin signaling and ecdysone response pathways regulate ovary development, the authors suggest that AgNPs may interfere with these pathways, resulting in the formation of small ovaries. Reproductive effects were also observed in zebrafish (*Danio rerio*) after exposure to AgNPs as apoptosis in the follicle cells surrounding the oocyte [35]. Thus, AgNPs may affect preovulatory follicle maturation and ovulation, impairing egg-laying capability genes [36,37].

### 2.4. Fecundity and Fertility

Sexual weakness, decreased birth rates, and infertility are potential consequences of environmental pollution. The progressive loss of fertility is directly correlated with environmental toxins internalized by both men and women [38]. Nanoparticles (NPs) can be ingested, inhaled, or dermally absorbed, then translocated to the circulatory system, accumulating in reproductive tissues and fetuses [39]. In *Drosophila*, fecundity is usually measured by counting the daily number of eggs laid by females over a 10-day period, while fertility is assessed by the progeny resulting from these eggs [40].

This study aimed to explore the effects of AgNP exposure on fecundity and fertility in *Drosophila*. The flies were exposed to AgNPs for one week and then divided into seven groups, each comprising 10 males and 10 virgin females, with three replicates per group. The first group included untreated flies, while the remaining six groups involved one or both genders exposed to 100 or 400 µg/mL of AgNPs (Figure S2).

**Figure 10:**
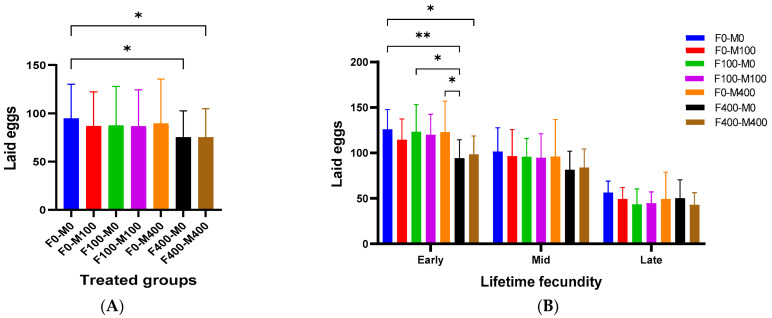
(**A**) Fecundity of different exposure groups treated for one week with AgNPs compared with non-treated ones. Multiple pairwise comparisons (Turkey’s test) were performed using ordinary one-way ANOVA. * *p* < 0.05. (**B**) Weekly fecundity of different treatment groups exposed for 1 week to AgNPs. Multiple pairwise comparisons (Turkey’s analysis) were performed using two-way ANOVA. * *p* < 0.05, ** *p* < 0.01. F, females; M, males; 0, nontreated flies; 100, flies treated with 100 µg/mL; and 400, flies treated with 400 µg/mL.

The results showed significant impacts of AgNP exposure on fly fecundity, especially in females exposed to 400 µg/mL, whether mated with untreated or treated (400 µg/mL) males (Figure 10A). The lifetime fecundity of *Drosophila* was observed to last for 21 days in all groups, except for the untreated group, which continued producing a few eggs for an additional five days. The average number of eggs laid during the lifetime fecundity period decreased from 596 eggs per female in the untreated group to 545 and 473 eggs per female in groups with both genders treated with 100 and 400 µg/mL of AgNPs, respectively. Fecundity over time was analyzed by extending the egg-laying period to 21 days, divided into three one-week subperiods. Fecundity disturbances were particularly noticeable during the first week in highly exposed flies (F400-M0 and F400-M400) (Figure 10B).

**Figure 11:**
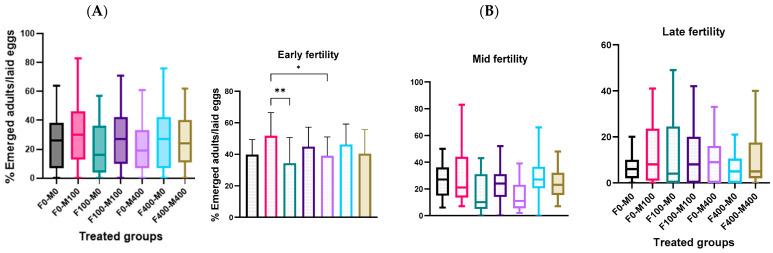
(**A**) Total fertility of *Drosophila* treated for 1 week to AgNPs. Multiple pairwise comparisons (Dunn’s test) were performed using Kruskal–Wallis test. (**B**) Weekly fertility of different *Drosophila* treated for 1 week with AgNPs. Multiple pairwise comparisons were performed using ordinary one-way ANOVA (Turkey’s test) or Kruskal–Wallis (Dunn’s test) test for parametric or non-parametric data, respectively. * *p* < 0.05, ** *p* < 0.01.

The impact of AgNP exposure on fertility was studied (Figure 11). While AgNP exposure did not change total fertility (Figure 11A), fertility disturbance was observed in the first week (Figure 11B). However, the exposure to AgNPs did not modify the normal ratio (♂♂/♀♀) in the offspring (Figures S4 and S5). Recent studies on *Drosophila* fecundity, after exposing larvae to AgNPs, reported decreased *Drosophila* fecundity, third-instar larvae weight, and rates of pupation and eclosion in a dose-dependent manner [41]. Although short exposure (3 days) to AgNPs did not influence egg-laying, a long exposure period (10 days) inhibited egg-laying capability in a dose-dependent manner [9]. However, it has been shown that nanomaterial uptake, whether by female or male flies, mediates developmental impairments like oogenesis disturbance and ovarian defect; reduces the egg length; delays egg chamber development, thereby decreasing fly fecundity; and delays the development of their offspring [42]. AgNP exposure altered ions homeostasis, reducing fertility [43]. In males, AgNPs induced spermatogenesis defects in the testis [8], and in exposed adults, AgNPs significantly decreased *Drosophila* fecundity, where larvae exposure decreased third-instar larvae weight and rates of pupation and eclosion in a dose-dependent manner [41].

### 2.5. Gene Expression Induction

**Figure 12:**
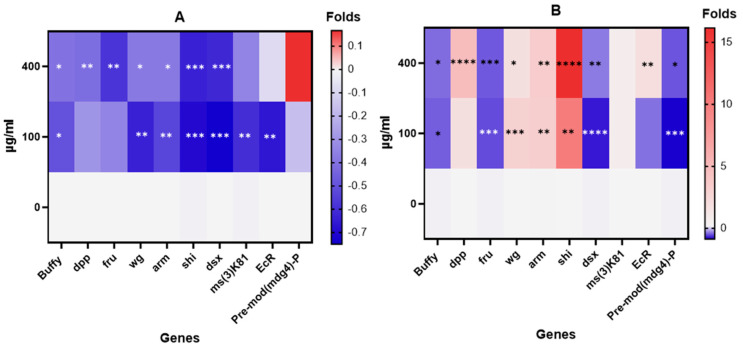
Heat map of gene expression profile for the 10 selected genes. Different behavior between males and females is observed. While females (**A**) show underexpression for all the cases, males (**B**) overexpress *arm*, *dpp*, *EcR*, *shi*, and *wg* genes. Multiple pairwise comparisons were performed using ordinary one-way ANOVA (Dunnett’s test). * *p* < 0.05, ** *p* < 0.01. *** *p* < 0.001, **** *p* < 0.0001.

Including gene expression studies in the battery of assays to determine the potential reproductive effects of AgNPs provides new mechanistic insights into the observed effects. Exposure to environmental agents can alter the balance in the expression levels of those genes involved in a common pathway, regulating the normal functionality of a defined system as the reproductive system. In such a context, changes in expression are considered a relevant marker of effect. Interestingly, the sensitivity of this target is higher than for other phenotypic biomarkers, giving special relevance to the inclusion of gene expression induction in the frame of the toxicity studies of environmental pollutants. The impact on the expression levels of the ten selected genes is shown in Figure 12. Gene expression levels in exposed flies are expressed as fold changes relative to those in untreated flies. In females, there is significant under-expression of all the selected genes except for *pre-mod(mdg-4)P*. In contrast, males exhibit significant over-expression in six out of ten genes (*arm*, *dpp*, *EcR*, *ms(3)K81*, *shi*, and *wg*). Supplementary Figure S6 presents a comparative analysis of gene expression between females and males. Interestingly, the AgNP exposure levels do not appear to be a factor significantly modulating the changes in expression levels.

Regarding the role of the different genes, *Armadillo* (*Arm*) plays a critical role in maintaining intestinal barrier function and regulating tissue regeneration [44]. In our study, the overexpression of *arm* was observed exclusively in exposed males, indicating compromised intestinal structural integrity and barrier function due to AgNP exposure. Recent studies have shown that contaminants like bisphenol A [45] and mercury [46] can alter the normal expression of *arm*, impairing intestinal barrier function. Additionally, the arm protein acts as a key effector in the *Wingless* signaling pathway. *Wingless* is the *Drosophila* homolog of mammalian Wnt oncoproteins, which play a central role in pattern generation during development. Notably, the suppression of *arm* activity in ovaries leads to defective eggs due to disrupted cell-to-cell adhesion [47]. *Buffy* is a key regulator of apoptosis [48]. Its ablation induces apoptosis, while its overexpression inhibits developmental programmed cell death [49]. In our study, *buffy* expression was downregulated in both females and males after AgNP exposure. Interestingly, *buffy* upregulation has been induced by nanoplastic exposure, linked to ovarian apoptosis and necrosis [11]. *Decapentaplegic (dpp)* regulates proper developmental processes in *Drosophila*, maintaining the anterior–posterior axis in the developing embryo and facilitating the formation of various tissues and organs in larvae and adult flies [50]. While *dpp* expression was not influenced in females after AgNP exposure, a substantial elevation was observed in males. Exposure to bisphenol A has been shown to cause underexpression of *dpp* in both males and females, adversely affecting the sexual behavior of Drosophila, especially in males [51]. *Doublesex (dsx)* is a sex-specific gene that regulates reproductive courtship in *Drosophila* [52]. In our study, *dsx* was under-expressed in both males and females after AgNP exposure, contrasting with the overexpression observed in males exposed to bisphenol A [51]. Interestingly, a large-scale functional screening using in vivo RNA interference in *Drosophila* detected that *doublesex/mab-3*-related transcription factor genes play an essential role in males [53], which supports our findings. The *ecdysone receptor (EcR*) gene in *Drosophila* adults governs lifespan, fertility, learning, behavior, and memory, as well as molting and metamorphosis [54]. Our study shows that AgNP exposure modulated *EcR* expression differently between sexes, causing underexpression in females and overexpression in males. Ecdysone is important in modulating the differential expression of some genes between males and females, highlighting the hormone’s relevance in sex differentiation [55]. AgNP exposure also affected ecdysone expression in *Chironomus riparius*, although sex differences were not determined [56]. In *Drosophila*, exposure to metals such as Cd and Hg caused developmental delays and mortality in a dose–response relationship, associated with significantly decreased *EcR* expression [57]. The *fruitless (fru)* gene, like *dsx*, plays a pivotal role in producing sexual dimorphism in *Drosophila*. Accordingly, the similar expression pattern shown by both genes is not surprising. It has been proposed that the products of *fru* and *dsx* genes maintain the sex-typical chromatin state at postembryonic stages, shape single neuron structures, and govern cell survival and death [58]. The *pre-mod(mdg4)-P* gene plays a crucial role in maintaining chromatin structure, nerve pathfinding, meiotic chromosome pairing, and apoptosis [59]. In our study, this gene is uniquely over-expressed in females and under-expressed in males. Exposure to heavy metals such as cadmium, copper, and lead has been shown to downregulate this gene in the adult head tissue of *Drosophila* [60]. Additionally, *pre-mod(mdg4)-P* overexpression has been observed after *Drosophila* exposure to polystyrene nanoplastics [11]. The male-specific *ms(3)k81* gene, essential for sperm chromatin organization during spermiogenesis, is specifically expressed in males [61]. Mutations in *ms(3)k81* cause male sterility without detectable morphological defects in testis and sperm development [62]. Despite its importance in male fertility, there are no studies linking changes in *ms(3)k81* expression to environmental toxicants. The *shibire (shi)* gene encodes a homolog of dynamin, crucial for endocytic membrane traffic [63]. It is highly expressed in early embryos, larval imaginal discs, and the gonads of both sexes [64]. *Shi* is also involved in maintaining actin cap integrity and sperm head compaction in *Drosophila* testis [65]. Our study found significant upregulation of *shi* in males, indicating a disturbance in spermatogenesis following AgNP exposure. However, no literature was found on the effects of environmental pollutants on *shi* expression. The *wingless (wg)* gene is essential for wing and haltere development, as well as segmentation and patterning during *Drosophila* development [47]. It regulates glycoproteins analogous to the *Wnt* gene in humans, influencing pathways involved in ovarian development, oogenesis, and early development [66]. Recent studies showed reduced *wg* expression in *Drosophila* larvae exposed to ZnO-NPs, correlating with aberrant phenotypes in their progeny [67]. In our study, *wg* expression was over-expressed in males and under-expressed in females, highlighting its role in reproductive traits.

In summary, differential expression patterns were detected between males and females for most of the selected genes. This supports the use of these genes as biomarkers for reproductive toxicity caused by AgNP exposure, potentially extending to other nanoparticles and organisms. It should be stated that the detection of differentially expressed genes represents just the first step associating exposure and reproductive toxicity. Deeper information on, e.g., pathways involved, would require complex transcriptomic analysis. Further, the role of a particular expressed gene would require functional analysis by using knockdown individuals.

## 3. Materials and Methods

### 3.1. AgNPs Obtention

AgNPs were purchased from nanoComposix (San Diego, CA, USA). According to the manufacturer, the standard properties are diameter (28 ± 4 nm), hydrodynamic size (63 nm), surface area (20.0 m^2^/g), ζ potential (−17 mV), and solvent (USP purified water). To further characterize AgNPs, transmission electron microscopy (TEM; Jeol 1400; Jeol Ltd., Tokyo, Japan) was used to show morphology, size, and degree of aggregation.

### 3.2. Experimental Design

The wild-type Canton-S strain of *Drosophila melanogaster* was used in all the experiments. The experimental design included treating *Drosophila* adults (males and females) with two different concentrations (100 and 400 µg/mL) of AgNPs, together with non-treated flies (0 µg/mL). Just after emergence, the flies were separated according to their sex and exposed separately (in groups of 50 flies). Peach–grape juice (with/without AgNPs) was used to feed adults. To proceed, 1 mL of juice (only juice for unexposed flies, and AgNPs dispersed in juice for treated flies) was used to wet the bottom of the clean sponge plug, normally used as a stopper for the flies’ vials. The last section explains how dispersions were prepared. In addition, a piece of paper was placed inside each vial to keep the flies’ mobility–relax regime. Flies were transferred every two days at intervals into new vials with new paper pieces and fresh wet plugs to reduce the flies’ mortality and keep a continuous feeding strategy. This type of exposure is a novelty to be pointed out. A representative image (Figure S1) is included in the Supplementary Material section to visualize how the exposure vials look.

### 3.3. AgNP–Juice Mix Preparation

Stock dispersions (40 mL) of AgNP–juice with different concentrations (100 and 400 µg/mL) were prepared following the Nanogenotox dispersion protocol [68] to guarantee good dispersions. Briefly, AgNP–juice solutions were sonicated (for 16 min) at 10% amplitude using an SSE-1 Branson sonicator. The sonicated solutions were immediately aliquoted into suitable tubes, frozen with liquid nitrogen for 10 s, and kept at −80 °C for further use.

### 3.4. Fecundity and Fertility

The newly emerged adults were collected and sorted into males and females. To confirm female virginity, first, all flies were removed from the vials, leaving only the pupae. The pupae vials were left for 8 h to produce new adults, which were collected, sorted according to their sex, and kept in new vials containing standard feeding media. These procedures were repeated until enough males and virgin females were obtained. At this point, males and females were divided into groups of 50 flies. After two days of emergence, groups of males and females were fed peach–grape juice (whether mixed with different AgNPs or without for the control flies). The treatment lasted for one week. At the end of the experiment, males and females were divided into seven groups; each group was constituted by 20 individuals (10 males with 10 females) in triplicate. The negative control group was established by mating both untreated males and females. The remaining six groups include one or both sexes exposed to 100 µg/mL or 400 µg/mL of AgNPs. A drawing showing the flies’ treatment scheme is provided in the Supplementary Materials (Figure S2). Mating groups were left for one day to adapt together and allowed to feed on a normal semi-solid medium at 24 ± 0.5 °C and 65 ± 5% relative humidity with a 12 h light/dark cycle. The feeding medium of *Drosophila* included 7 mg/mL agar-agar, 1.5 mg/mL NaCl, 87 mg/mL fresh yeast, 123 mg/mL corn flour, and 2 mg/mL of methylparaben and 0.002% *v*/*v* propionic acid as preservative ingredients. Methylparaben is dissolved in ethanol (125 mg/mL). Normal medium was mixed with black carbon to increase the contrast and optimize the counting of laid eggs, On the morning of every day, the flies were transferred into new vials containing new black medium, while their production of eggs was counted (fecundity) and kept in incubation conditions until adults emerged. The last procedure was repeated every day, for 21 days, which agreed with the laying period. No eggs were observed after this laying period except for non-treated flies, producing a few eggs for six days more. Once the emerged adults were counted (fertility), males and females were separated and counted to know each gender percentage. Fecundity was determined as the number of eggs produced daily for each group, while fertility was calculated as the percentage of emergency adults corresponding to laid eggs. To address the impact of AgNP exposure on the ovary size, AgNP-treated females were dissected, and their ovaries (100 ovaries for each group) were photographed and measured with the ImageJ program.

### 3.5. Ag Concentration Quantification with ICP-MS

The inductively coupled plasma mass spectrometry (ICP-MS) technique was applied to evaluate the Ag content in males, females, and their reproductive organs, as well as in testes and ovaries after exposure to 0, 100, and 400 µg/mL AgNPs. To determine the ability of AgNPs to be transferred into fly gametes, Ag concentrations were analyzed in eggs after one week of exposure to different AgNP concentrations. The egg Ag content after cleaning the midgut from AgNPs was determined in three scenarios (i) egg collection starts just after 1-week exposure, (ii) egg collection starts 8 h after 1-week exposure (during the 8 h, feed on non-treated juice in the sponge plug), and (iii) egg collection starts 1 day after 1-week exposure (during this day, adults feed on non-treated juice in the sponge plug). During the oviposition period, adults feed on a standard medium. All egg samples were kept at −80 °C until ICP-MS quantification analysis. The experiment was quadruplicated, and unexposed egg samples were used as the baseline control. Before performing ICP-MS, all samples (males, females, testes, ovaries, and eggs) were incubated at 70 °C overnight to dry according to our previous protocol [69]. To proceed, samples were weighed and digested with a mixture of hydrofluoric acid and HNO_3_ in a microwave oven at 270 °C for 1 h under high pressure. Then, the digested samples were diluted in HNO_3_ (1% *v*/*v*) with a final volume of 10 mL and weight. Finally, Ag concentrations were determined using an Agilent Model 7900 Inductively Coupled Plasma Mass Spectrometer device.

### 3.6. AgNP Internalization

The internalization and distribution of AgNPs (400 µg/mL) inside the different body compartments, including the gastrointestinal tract, hemolymph, ovaries, testes, and even feces, after exposure lasting for one week was investigated. Transmission and confocal microscopy methods were recruited to verify this goal.

### 3.7. TEM Investigation

- *Ultrathin sections*. The presence of AgNPs in various body tissues, including reproductive organs of *Drosophila* adults (Figure S3), was determined following our previously described protocol [70]. Adult samples (intestine, testes, and ovaries) were dissected in buffer solution (PB; 0.1 M, pH 7.4) and immediately kept in a fixation solution (0.15 M phosphate buffer containing 4% paraformaldehyde and 1% glutaraldehyde, pH 7.4). Then, the samples were transferred into TEM services for further manipulation. The samples were post-fixed and stained with a mixture of 0.8% (*w*/*v*) potassium hexacyanoferrate and 1% (*w*/*v*) osmium tetroxide for 2 h in a cold temperature environment (4 °C). To discard staining excess, samples were washed with deionized water, followed by a graded series of acetone for dehydration and embedded in Eponate 12TM resin (Ted Pella Inc., Redding, CA, USA). The resin-bound samples were allowed to polymerize at 60 °C for 48 h to be ready for sectionalization. A diamond knife (450, Diatome, Biel, Switzerland) was used to obtain ultrathin sections. The last preparation step included sections mounting upon non-coated copper grids, which contrasted with uranyl acetate for 30 min, followed by Reynolds lead citrate (5 min). A TEM (HITACHI H-7000, Jeol Ltd., Tokyo, Japan), 125 kV) device was used to examine AgNPs’ distribution into the different compartments of the gut, including the lumen, the symbiotic bacteria of the gut lumen, and enterocytes. The tissues of ovaries and testes were also investigated to examine AgNP internalization in reproductive organs.
- *Loaded grid hemolymph*. Adults were dissected to detect AgNPs in the flies’ hemolymph. The obtained hemolymph was mounted upon copper girds, dried, and investigated with TEM.
- *Digested feces*. Pieces of paper containing adult exposed feces were collected, stirred at 700 rpm for 1 h, and filtered to discard paper fragments. The filtered solutions were aliquoted into 10 mL tubes and centrifuged at 10,000 rpm for 30 min. The supernatants were removed except for the bottom 1 mL of each tube, which was collected and placed in one tube and centrifuged again. Drops of the bottom solution “after removing the supernatant” were deposited on a TEM grid for further investigations.

### 3.8. Confocal Microscopy

Organ samples (crop, intestine, Malpighian tubes, hemocytes, testes, and ovaries) from exposed adults were dissected in 1% PBS and transferred to the confocal services for further manipulation. Samples were stained with Hoechst 33342 (excitation of 405 nm and emission collected at 415–503) and Cellmask (excitation of 633 nm and emission collected at 645–786) to visualize the nuclei and cellular membranes, respectively. The presence of AgNPs in the different tissues was determined via reflection properties using a confocal microscopy (Leica TCS SP5, Leica Microsystems GmbH, Mannheim, Germany) device.

### 3.9. Molecular Response (Real-Time PCR)

Changes in gene expression levels might reflect physiological alterations in cellular homeostasis at the molecular level. Thus, changes in the expression of several reproductive-related genes were evaluated in female and male flies after one week of exposure to AgNPs (0, 100, and 400 µg/mL). The investigated genes include *arm*, *Buffy*, *dpp*, *dsx*, *EcR*, *fru*, *mdg4*, *ms*, *shi*, and *wg.* The forward and reverse primers are indicated in Table S1 and were designed based on the National Center for Biotechnology Information NCBI and the work of Tu et al. [11]. The housekeeping reference *actine 5C* (*Act5C*) gene was used for gene normalization. This gene expression study was made according to our previous protocol [71]. RNA was extracted from homogenized adults (males or females) in TRItidy G™ (PanReac AppliChem), quantified with a NanoDrop 1000 spectrophotometer, and treated with RNase-free DNase kits to avoid DNA contamination. cDNA (100 ng/µL) was obtained via reversely transcribed RNA samples. Finally, cDNA samples were quantitatively polymerized by real-time PCR using a LightCycler^®^ 480 SYBR Green I Master (Roche, Mannheim, Germany). The RT-PCR cycling included pre-incubation (95 °C for 5 min, one cycle), amplification (45 cycles of 95 °C for 10 s, 63 °C for 15 s, 72 °C for 25 s, and 78 °C for 5 s), and one cycle for each melting curve and cooling. Cycle threshold (Ct) data were calculated with the LightCycler software (3.0 version). The gene expression values of exposed flies were normalized and represented in folds and compared with non-treated values.

### 3.10. Statistical Analysis

Data were analyzed using GraphPad Prism 9 software (GraphPad Software Inc., La Jolla, CA, USA). The normal distribution of data was checked using the Shapiro–Wilk test. Correlation (Pearson r) and linear regression analyses were performed to highlight potential relationships with different variables. The one-way ANOVA or two-way ANOVA (parametric tests) with Tukey’s multiple comparison test was applied for normally distributed data. The Kruskal–Wallis (non-parametric test) with Dunn’s multiple comparison test was used for skewed normality data. Normally distributed data were represented as mean ± standard deviation, while the median and interquartile range (box and violin) represented the nonparametric data. *p* values, * *p* ≤ 0.05, ** *p* ≤ 0.01, *** *p* ≤ 0.001, and **** *p* ≤ 0.0001 were used to define statistical significance between independent variables or in comparison with controls.

## 4. Conclusions

By administering AgNPs mixed with peach–grape juice, we demonstrate that ingested AgNPs are widely distributed across various body organs, including reproductive tissues such as testes, ovaries, sperm, and eggs. This illustrates the capability of AgNPs to permeate the gastrointestinal barrier to reach those organs. Importantly, through the intestinal tract, AgNPs retained their original diameter, indicating their stability within biological tissues and fluids. Our ICP-MS analysis confirmed the bioaccumulation of AgNPs in reproductive organs and their transfer to gametes (eggs), revealing temporal changes in Ag accumulation in eggs following the cessation of AgNP exposure. Among the organs examined, testes emerged as the primary target of AgNP accumulation, raising concerns about potential fertility impacts in males exposed to these nanoparticles. Additionally, AgNP exposure results in a reduction in ovary size, adversely affecting fecundity. However, no significant effects on overall fertility and sex ratio were observed. Interestingly, we observed alterations in the expression levels of several genes associated with reproduction, development, and apoptosis, which indicate the reproductive toxicity of nanomaterials. Given the importance of assessing reproductive toxicity, our findings support the potential use of *Drosophila* in New Approaches Methodologies (NAMs) in regulatory science to identify agents and exposures that may pose reproductive hazards.

## Data Availability

Data and materials will be available upon request.

## Supplementary Materials

The following supporting information can be downloaded at: https://www.mdpi.com/article/10.3390/molecules29235802/s1, Figure S1. Drosophila feeding design. Peach-grape juice (A), flies (B), wet sponge plug (C), and flies inside tubes closed with juice-wetted sponge plug. Paper pieces were placed inside tubes to regulate fly activities; Figure S2. Schematic figure showing the various organs where AgNP internalization was investi-gated; Figure S3. Schematic figure of fecundity and fertility experiment of various AgNPs-exposed groups of Drosophila; Figure S4. Total F/M ratio of different *Drosophila melanogaster* groups treated for one week with AgNPs. Multiple pairwise comparisons (Dunn’s test) were performed using Kruskal-Wallis; Figure S5. Weekly F/M ratio of different *Drosophila melanogaster* treated for one week with AgNPs. Multiple pairwise comparisons were performed using Ordinary one-way ANOVA (Turkey’s test) or Kruskal-Wallis (Dunn’s test) for parametric or non-parametric data, respectively; Figure S6. The molecular response to AgNPs exposure. Multiple pairwise comparisons (Turkey’s test) were performed using Ordinary one-way ANOVA. * *p* < 0.05, ** *p* < 0.01, *** *p* < 0.001, and **** *p* < 0.0001; Table S1. Gene primers of *D. melanogaster* used in quantitative RT-PCR. Primers were designed according to the National Center for Biotechnology Information (NCBI) (https://www.ncbi.nlm.nih.gov/) and Tu et al. (2023).
