# Supplementary material for: Reproductive Toxicity of Nanomaterials Using Silver Nanoparticles and Drosophila as Models
Source: Molecules. 2024 Dec 9;29(23):5802. doi: 10.3390/molecules29235802 (PMC11643907; doi:10.3390/molecules29235802)
Supplement: Supplementary file 1 [file molecules-29-05802-s001.zip › molecules-3324305-supplementary.pdf]

Reproductive toxicity of nanomaterials using silver nanoparticles and *Drosophila* as models

Mohamed Alaraby[1,2,*], Doaa Abass[1,2], Javier Gutiérrez[1], Antonia Velázquez[1], Alba Hernández[1,*]Ricard Marcos[1,*]

[1]*Group of Mutagenesis, Department of Genetics and Microbiology, Faculty of Biosciences, Universitat Autònoma de Barcelona, Cerdanyola del Vallès, Spain.*

[2]*Zoology Department, Faculty of Science, Sohag University (82524), Sohag, Egypt.*

SUPPLEMENTARY MATERIAL

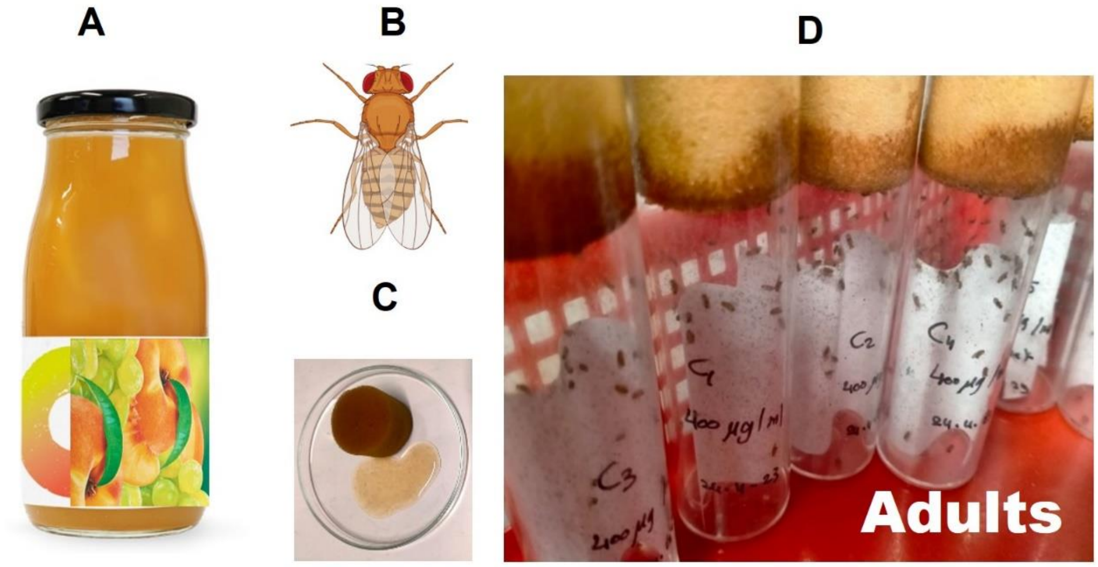

Figure S1. Drosophila feeding design. Peach-grape juice (A), flies (B), wet sponge plug (C), and flies inside tubes closed with juice-wetted sponge plug. Paper pieces were placed inside tubes to regulate fly activities.

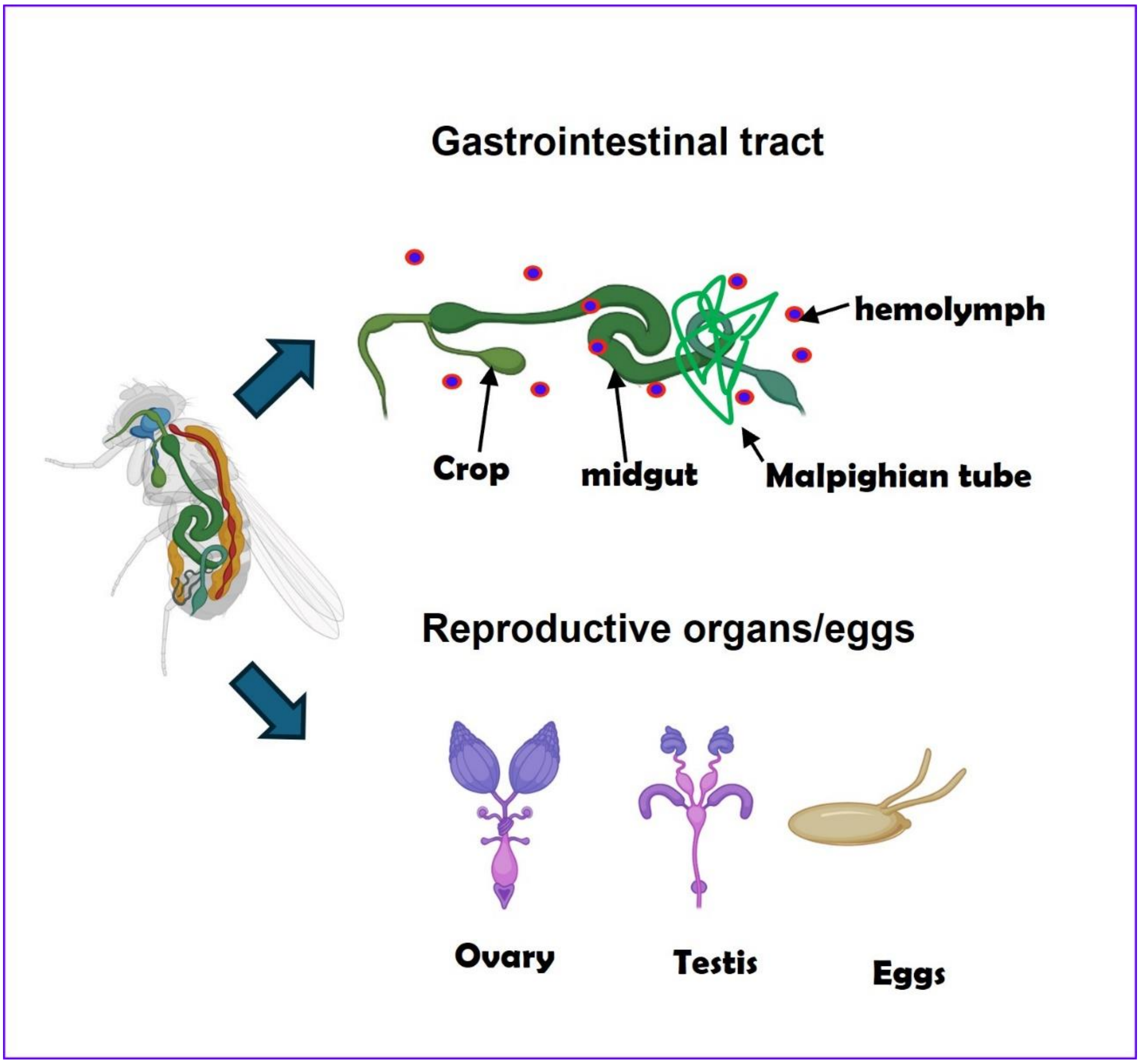

Figure S2. Schematic figure showing the various organs where AgNP internalization was investigated.

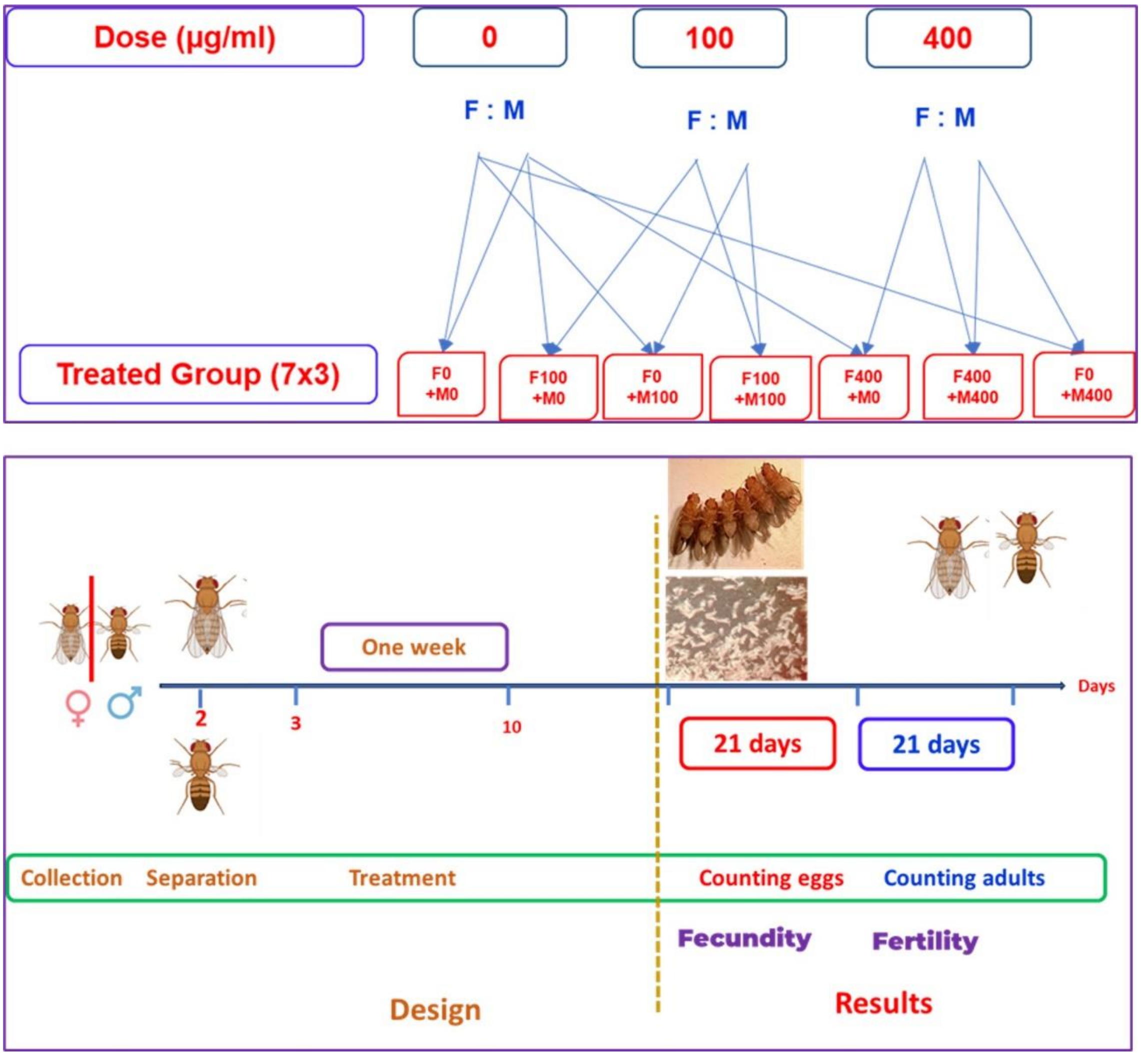

Figure S3. Schematic figure of fecundity and fertility experiment of various AgNPs-exposed groups of *Drosophila.*

Table S1. Gene primers of *D. melanogaster* used in quantitative RT-PCR. Primers were designed according to the National Center for Biotechnology Information (NCBI) (https://www.ncbi.nlm.nih.gov/) and Tu et al. (2023).

No.	Gene	Forward Primer (5′-3′)	Reverse Primer (5′-3′)	Function
1	Transcript variant B (Act5C) NM_078497.4	GATGAAGATCCTGACCGAGC	TGTAGAAGGTGTGGTGCCAG	Housekeeping actine 5C (Act5C), as a reference gene, was used for gene normalization
2	Buffy (Buffy) NM_078978.2	TGCATCCCCGGATATACAACG	CCAAAAGCAAGCTCACAGCAT	Including ectopic germ cell programmed cell death, apoptosis
3	Pre-mod(mdg4)-P	GCTTCCCTGGAAGAAAGATAGG	CTGAAGACCTACTGGATATGCTCC	Gamete generation (cell proliferation)
4	Decapentaplegic (dpp) NM_057963.5	GAGGCGAAAGATCGCTGGTT	GAGAACGAGAAACGACGGGA	Including animal organ development; gamete generation
5	Fruitless (fru) NM_079673.3	AGACGGACAATATCGACGGC	ATGGTACTCGCGTTTCAGGG	Mating behaviour, and neuron development
6	Wingless (wg) NM_078778.5	GTGAAGTGCAAGCTGTGTCG	AGGGGCGGTTAGTCGTATCT	Animal organ development, open tracheal system development
7	Armadillo (arm) NM_057317.4	CGGTTATCTGTTCTGCGCTC	CTGGGCTGGCATGTAACTCA	Generation of neurons, morphogenesis of an epithelium
8	Shibire (shi) NM_206742.1	TGCAGTGAAGTAAAGCCAGC	GAACGCATCTTGCAGCTTGT	Endocytosis, germ cell development
9	Ecdysone receptor, (EcR) NM_001169590.2	ATGGGGTGCGAGTGAAAGAC	CACTCGACTTGGGTCCGAAA	Animal organ development; embryonic development via the syncytial blastoderm, and instar larval or pupal development
10	Doublesex (dsx) NM_169202.1	CAGTGCATCCAGCAGTCGAG	CGATCGATGCCAGCAAACTG	Imaginal disc-derived genitalia development
11	Male sterile (3) K81 (ms(3)K81) NM_143253.2	CGCCAACGTATACTAGCCCC	ACCGCAGCTCCGAGTTTATC	Male sterile responsible for produce mature sperm or mate properly. The m(3) K81 mutation lead to developmental arrest after sperm entry into the egg

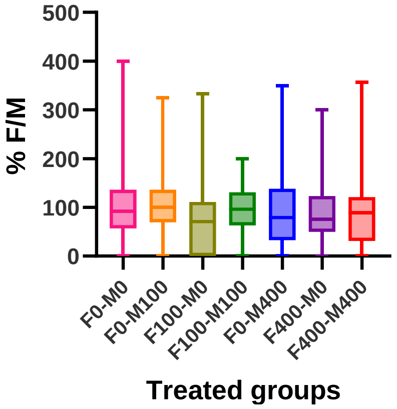

Figure S4. Total F/M ratio of different *Drosophila melanogaster* groups treated for one week with AgNPs. Multiple pairwise comparisons (Dunn's test) were performed using Kruskal-Wallis.

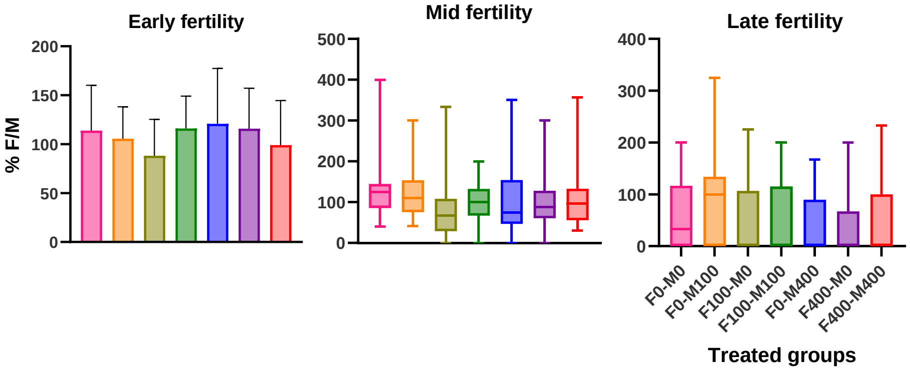

Figure S5. Weekly F/M ratio of different *Drosophila melanogaster* treated for one week with AgNPs. Multiple pairwise comparisons were performed using Ordinary one-way ANOVA (Turkey's test) or Kruskal-Wallis (Dunn's test) for parametric or non-parametric data, respectively.

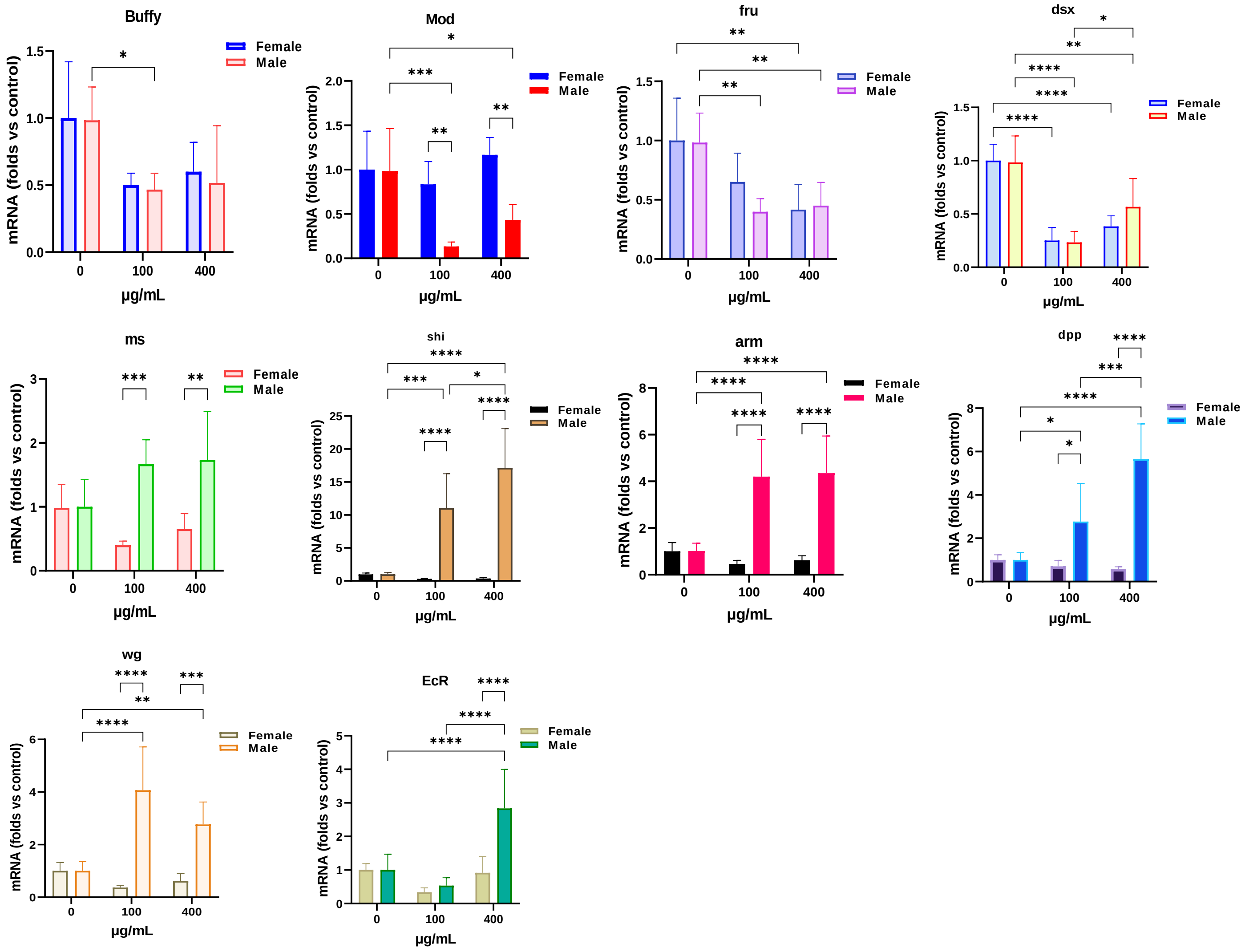

Figure S6. The molecular response to AgNPs exposure. Multiple pairwise comparisons (Turkey's test) were performed using Ordinary one-way ANOVA. **P*<0.05, ***P*<0.01, ****P*<0.001, and ****P<0.0001.
